# Diseases of the Nervous System

**Published:** 1899-03

**Authors:** 


DISEASES OF THE NERVOUS SYSTEM. A hand-book for Students and Practitioners. By CHARLES E BEEVOR, M. D., London, F. R. C. P. Physician to the National Hospital for the Paralysed and Epileptic, etc. Duodecimo, pp. xv.—432. Illustrated. Philadelphia: P. Blakiston, Son & Co., 1012 Walnut street. 1898.

The author has succeeded admirably in presenting the subject of nervous diseases in a clear and concise manner. He has learned to condense without omitting anything that is necessary to a complete presentation of his subject. In the chapter on general anatomy and physiology, he has chosen to omit the minute anatomy of the medulla, but refers the reader to the anatomy in the larger text-books. This is wise as there is no advantage in burdening a handbook on diseases with anatomical minutiæ, which can be found in all the late treatises.

The majority of the illustrations are original and are founded on the experimental work of Horsley and the author, and on typical clinical cases.

His description of diseases is always graphic, concise and clear. We do not think he gives sufficient value to ocular defects as etiological factors of functional nervous diseases. For instance, in migraine he says exciting causes are overwork and worry, prolonged using the eyes in over-microscopical work and study. This is clearly understating the case. In epilepsy no mention is made of the possibility of errors in refraction, or muscular defects being causes. In the treatment of diseases he is no hobbyist, but gives the reader a choice of methods. He differs from the Americans in his advice concerning trephining for epilepsy. In this country we have come to advise operation only in recent cases or in cases in which we find external evidence of skull injury. We have come to this conclusion as a result of repeated failures to relieve chronic cases. Beevor says: "Surgical treatment should be employed in cases where the fits are very frequent and are not kept under the bromides and begin with a definitely localised warning as tingling or spasm of the thumb."

He advises the removal of that piece of the cortex which by faradic stimulation is found to produce the movement with which the fit begins. He has no objection apparently to operating upon old cases.

As to the style of nomenclature it is purely conservative. None of the new anatomical terms adopted in our later text-books in America are used by him. Aside from this thoroughly English conservative characteristic, we have nothing but praise for the book.

